# Sensitivity of Intervertebral Disc Finite Element Models to Internal Geometric and Non-geometric Parameters

**DOI:** 10.3389/fbioe.2021.660013

**Published:** 2021-06-17

**Authors:** Yuekang Du, Saman Tavana, Tamanna Rahman, Nicoleta Baxan, Ulrich N. Hansen, Nicolas Newell

**Affiliations:** ^1^Biomechanics Group, Department of Mechanical Engineering, Imperial College London, London, United Kingdom; ^2^Biological Imaging Centre, Central Biomedical Services, Imperial College London, London, United Kingdom

**Keywords:** intervertebral disc, finite element model, magnetic resonance imaging, cohesive elements, sensitivity

## Abstract

Finite element models are useful for investigating internal intervertebral disc (IVD) behaviours without using disruptive experimental techniques. Simplified geometries are commonly used to reduce computational time or because internal geometries cannot be acquired from CT scans. This study aimed to (1) investigate the effect of altered geometries both at endplates and the nucleus-anulus boundary on model response, and (2) to investigate model sensitivity to material and geometric inputs, and different modelling approaches (graduated or consistent fibre bundle angles and glued or cohesive inter-lamellar contact). Six models were developed from 9.4 T MRIs of bovine IVDs. Models had two variations of endplate geometry (a simple curved profile from the centre of the disc to the periphery, and precise geometry segmented from MRIs), and three variations of NP-AF boundary (linear, curved, and segmented). Models were subjected to axial compressive loading (to 0.86 mm at a strain rate of 0.1/s) and the effect on stiffness and strain distributions, and the sensitivity to modelling approaches was investigated. The model with the most complex geometry (segmented endplates, curved NP-AF boundary) was 3.1 times stiffer than the model with the simplest geometry (curved endplates, linear NP-AF boundary), although this difference may be exaggerated since segmenting the endplates in the complex geometry models resulted in a shorter average disc height. Peak strains were close to the endplates at locations of high curvature in the segmented endplate models which were not captured in the curved endplate models. Differences were also seen in sensitivity to material properties, graduated fibre angles, cohesive rather than glued inter-lamellar contact, and NP:AF ratios. These results show that FE modellers must take care to ensure geometries are realistic so that load is distributed and passes through IVDs accurately.

## Introduction

Intervertebral discs (IVDs) lie between vertebra in the spine and act to distribute loading while allowing the spine to bend and flex (Bogduk, 2005; Adams and Roughley, 2006). The IVD consists of the anulus fibrosus (AF), nucleus pulposus (NP) and the endplates that enclose the NP and AF above and below. As the IVD degenerates, its height reduces (Adams et al., 1987), the relative ratio of NP to AF cross-sectional area decreases (Adams et al., 1996), and the stiffness of individual components increases (Iatridis et al., 1998, 1999; O’Connell et al., 2009). Previously, these changes have been investigated using experimental techniques, however, it is challenging to measure stresses and strains within the IVD without disrupting it, and therefore, there has been an increasing trend towards the use of FE models for investigations of this kind.

Previous FE studies on human lumbar spines have explored the effect of altering geometric features of the IVD (Robin et al., 1994; Natarajan and Andersson, 1999; Noailly et al., 2007; Meijer et al., 2011; Niemeyer et al., 2012). All of these studies have found the disc height significantly affects the response of the IVD, and some have shown that NP position (Noailly et al., 2007), and endplate width and depth (Natarajan and Andersson, 1999; Meijer et al., 2011; Niemeyer et al., 2012) are important factors. Similarly, the nature of the contact between lamella in the AF in FE models has been shown to affect model response (Adam et al., 2015), and a method of using cohesive contact between lamella has been proposed by Mengoni et al. (2015). Recent FE studies on bovine (Mengoni et al., 2017), and human discs (Yang et al., 2019), have demonstrated the importance of the NP:AF ratio, few studies have investigated the effect of changes to the internal IVD geometry. This is likely due to the challenges associated with defining the boundary between the NP and AF. Recent advances in magnetic resonance imaging (MRI) allow accurate internal geometries to be identified (O’Connell et al., 2011; Tavana et al., 2020, 2021). In some cases, these geometries are substantially different to those that are used in current FE models, and the effect of these inaccuracies has not previously been investigated.

The aim of this study is to use high resolution MRI scans, and FE models to investigate the effect of altered geometries both at the boundary between the NP and the AF, and at the endplates on the response of the IVD. Specific aims include quantifying the effect of altered geometries on IVD;

(a) stiffness and strain distributions
(b) sensitivity to altered material properties
(c) sensitivity to graduated fibre bundle angles (increasing from outer to inner AF)
(d) sensitivity to modelling inter-lamellar behaviour with cohesive contact
(e) sensitivity to altered NP:AF ratio.

## Materials and Methods

For this study, a vertebral body—disc—vertebral body specimen was dissected from the most caudal disc of a fresh-frozen bovine tail acquired from a local butcher. Soft tissue was removed before being scanned on a 9.4 T MRI scanner (Bruker BioSpec, Ettlingen, Germany) equipped with a volume RF resonator [T_2_-weighted RARE sequence, coronal plane, resolution = (90 × 90) μm^2^, slice thickness = 800 μm, 17 min scan time], such that internal geometries could be acquired.

### Experimental Data

Experimental data, against which the FE models could be compared was obtained from previous literature (Newell et al., 2017). During these experiments ten bovine IVDs were axially compressed to 15% strain at a range of strain rates and the force-displacement response of each sample was recorded. For comparison purposes only data from mid-range strain rates (0.1/s) were used in this study.

### FE Model Development

**FIGURE 1:**
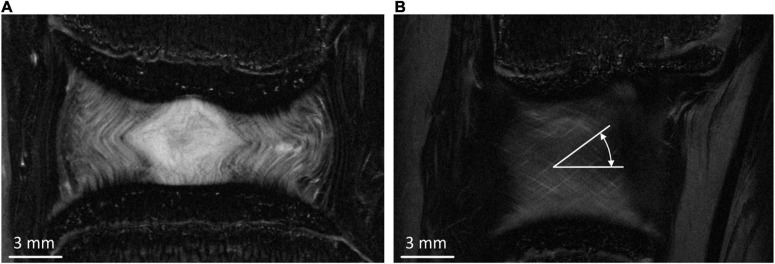
**(A)** Middle coronal slice of the MRI of the bovine IVD. **(B)** Typical peripheral MRI slice from which fibre bundle angles were measured.

Non-linear, implicit, axisymmetric FE models using Marc (v2017, MSC Software, California, United States) were developed based on measurements from the mid-coronal slice of the high-resolution MRI images (Figure 1A).

### Geometry

**FIGURE 2:**
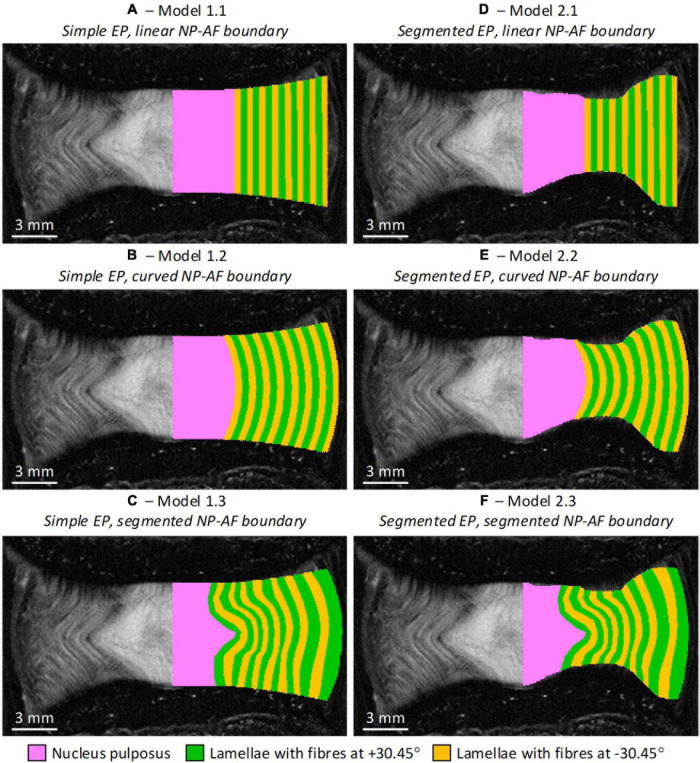
Initial geometry of the six axisymmetric FE models overlaid on the MRI slice from which the geometries were obtained. Note the axis of symmetry in the models is along the mid-sagittal plane of the IVD. **(A)** Model 1.1—simple endplates, linear NP-AF boundary, **(B)** Model 1.2—simple endplates, curved NP-AF boundary, **(C)** Model 1.3—simple endplates, segmented NP-AF boundary, **(D)** Model 2.1—segmented endplates, linear NP-AF boundary, **(E)** Model 2.2—segmented endplates, curved NP-AF boundary, **(F)** Model 2.3—segmented endplates, segmented NP-AF boundary. Note the fibre bundle angles are relative to the transverse plane.

Six FE models were developed, with two variations of endplate geometry (simple and segmented), and three variations of NP-AF boundary (linear, curved, and segmented) (Figure 2). Since the bovine tail disc is almost perfectly round all FE models were axisymmetric (Adam et al., 2015). The simple endplate geometries (Model 1.1, 1.2, and 1.3) had central and peripheral IVD heights measured from the mid-coronal MRI slice but a smooth curve between these two points. The segmented endplate geometries (Model 2.1, 2.2, and 2.3) were obtained from the mid-coronal MRI slice using Mimics (v16, Materialise, Leuven, Belgium) but also had central and peripheral IVD heights that matched those of the simple endplate geometry models. The linear NP-AF boundary models had a vertical, linear boundary between the NP and AF, while the curved NP-AF boundary models had a quadratically polynomial boundary. Both the linear, and curved NP-AF boundary models had a NP:AF ratio determined from measurements at the mid-height of the mid-coronal slice of the IVD. As with the endplates, the segmented NP-AF boundary was obtained from the mid-coronal MRI slice using Mimics.

The AF was modelled with rebar elements to represent the collagen fibre bundles, surrounded by non-linear solid quadrilateral elements to represent the AF ground matrix. For the models with segmented NP-AF boundary, each lamella layer was segmented, while the number of rebar layers for the linear and curved models was set to the number of layers that could be identified on the mid-coronal slice of the MRIs. The NP was modelled with non-linear solid triangular elements. For this study endplates and vertebral bodies were assumed to be rigid as the effect of the endplates, and VBs on the behaviour of bovine IVD FE models has been found to be negligible (<2.5% difference in the peak force; Newell et al., 2017). A convergence study was performed on each specimen to ensure that the mesh density was sufficient.

### Material Properties

The properties of each material used in the models are shown in Table 1. The AF matrix and the NP were assigned non-linear hyperelastic material properties (Mooney-Rivlin). The strain energy function for this material model is shown in Eq. 1, where W is the strain-energy density function, I_1_ and I_2_ are strain invariants, and C_10_ and C_01_ are material constants (Mooney, 1940):

**TABLE 1:** Baseline material properties for components of each of the FE models.

**Component**	**Material model**	**Material parameters (MPa)**	**References**
Collagen fibre bundles	Linearly elastic	415	Newell et al., 2017
AF ground substance	Hyperelastic	*C*_10_ = 0.7, *C*_01_ = 0.2	Natali and Meroi, 1990
Nucleus pulposus	Hyperelastic	*C*_10_ = 0.07, *C*_01_ = 0.02	Adam et al., 2015

(1) $$W=C_{10}\,\left(I_{1}-3\right)+C_{01}\,\left(I_{2}-3\right)$$

The AF fibre bundles were modelled using tension only rebar elements with a Young’s modulus (YM) of 415 MPa which was obtained from the results of an optimisation study (Newell et al., 2017). The fibre bundles were aligned at ±30.45° to the transverse plane which was an average of six measurements taken from different coronal MRI slices at regular intervals from the inner to outer AF (Figure 1B). The cross-sectional area of each fibre bundle was set to be 3.212 × 10^–2^ mm^2^, and spacing of the bundles was set to 4.35 bundles/mm (Marchand and Ahmed, 1990; Adam et al., 2015). The bulk modulus of the NP and AF ground matrix was set at 2,000 MPa to ensure near incompressibility.

### Boundary Conditions

Replicating the experimental setup described by Newell et al. (2017), the inferior boundary of the IVD was fixed, and a displacement of 0.86 mm, which corresponded to a central disc axial strain of 15%, was applied to the superior boundary of the IVD. Since the models with segmented endplates had a shorter overall (or average) disc height (eventhough the central disc heights were kept the same), and the displacement of 0.86 mm was applied to all models, the models with segmented endplates were subjected to higher overall (or average) axial strains. Since the model was axisymmetric, nodes along the axis of symmetry were fixed in the radial direction.

### Sensitivity Study

The influence of the AF C_10_, AF C_01_, fibre bundle YM, and fibre bundle angle values were investigated by varying their baseline value by ±20% and observing the effect on the peak force.

### Graduated Fibre Bundle Angle

A number of previous FE studies have modelled fibre bundles with a constant orientation from outer to inner AF (Shirazi-Adl et al., 1986; Marchand and Ahmed, 1990; Natali and Meroi, 1990; Adam et al., 2015; Newell et al., 2017). However, in human IVDs fibre bundle angle has been reported to vary linearly from 28(outer) to 45(inner) in the radial direction relative to the transverse plane (Cassidy et al., 1989), and has been included in a number of other FE studies (Ayturk and Puttlitz, 2011; Schmidt et al., 2012). From the MRI scans, fibre bundle angles of 27.4° and 37.4° were measured at the outer most layer and inner most layer, respectively. In order to understand the effect of including a variation in fibre bundle angle, each layer of fibre bundles were assigned material properties individually in the model. The outer most layer was assigned 27.4° and the inner most layer was assigned 37.4°, while intermediate angles were varied linearly between these two angles.

### Modelling Inter-lamellar Behaviour With Cohesive Contact

Interactions between lamellar in the AF were modelled using cohesive elements which allows the interfaces to be described by traction-separation laws. Normal cohesive stiffness (Knn = 1.18 MPa/mm), and tangential cohesive stiffness (Ktt and Kss = 1.31 MPa/mm) values were taken from Mengoni et al. (2015) who derived values using an optimisation algorithm and simulations of tension experiments where ovine AF samples were loaded radially. A stiffening factor in compression was assigned to ensure penetration between elements in adjacent lamellae was minimal. Preliminary investigations showed that a factor of 100,000 was sufficient to ensure that ±20% change resulted in a less than 2% change in peak force.

### NP:AF Ratio

**FIGURE 3:**
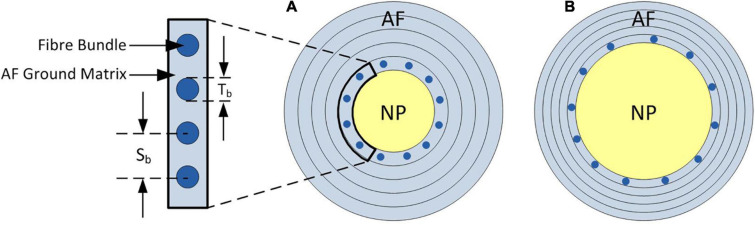
Schematic of axial cross-section of the IVD demonstrating the change in fibre bundle spacing required to allow a fair comparison between models when adjusting the NP:AF ratio. **(A)** Shows a baseline NP:AF ratio with a cut out showing the fibre bundle thickness (Tb), and interbundle spacing (Sb), **(B)** shows an increased NP:AF ratio with larger interbundle spacing. Bundles are only shown in the inner most lamella in both schematics, with a total of 12 bundles shown in both **(A,B)**, although larger interbundle spacing in **(B)** resulting in the same fibre bundle volume as that shown in **(A)**. Note these schematics are not to scale.

The NP:AF ratio was doubled (increasing the NP radius compared to the AF width but keeping the overall IVD width constant) in each of the models to investigate the effect of NP:AF ratio on the mechanics of the disc. Doubling the ratio ensured that there was a clear divergence from the baseline geometry while keeping within the physiological bounds of reported NP:AF ratios (O’Connell et al., 2007; Adam et al., 2015; Newell et al., 2017). In all baseline models the number of rebars/mm was defined based on measurements from Marchand and Ahmed (1990) (4.35 bundles/mm = 0.22 mm interbundle spacing) (Figure 3A). When increasing the NP:AF ratio care was taken to ensure that the total number of fibre bundles, was the same between the baseline, and adjusted NP:AF ratio models (Figure 3). This was achieved by calculating the difference between the circumference of each lamella of the baseline and the altered NP:AF ratio models using the horizontal distance from each lamella to the axisymmetric axis. The fibre bundle spacing was then adjusted to ensure each new model had the same fibre volume as the baseline models (Figure 3).

## Results

Taking measurements from the MRIs, the sample had a central disc height of 8.41 mm, sagittal plane width of 25.56 mm, a coronal plane width of 24.36 mm, and an area of 489.30 mm^2^. A convergence study was performed on each specimen to ensure that the mesh density was sufficient. This involved subdividing the number of elements and comparing the peak force obtained using the original mesh with that obtained with the subdivided mesh. If peak forces were within 1% the original mesh was considered converged, otherwise further subdivisions were performed until consistent (within 1%) peak forces were found. This resulted in models having an average of 1,163 ± 469 elements.

### Stiffness and Stress Distributions

**FIGURE 4:**
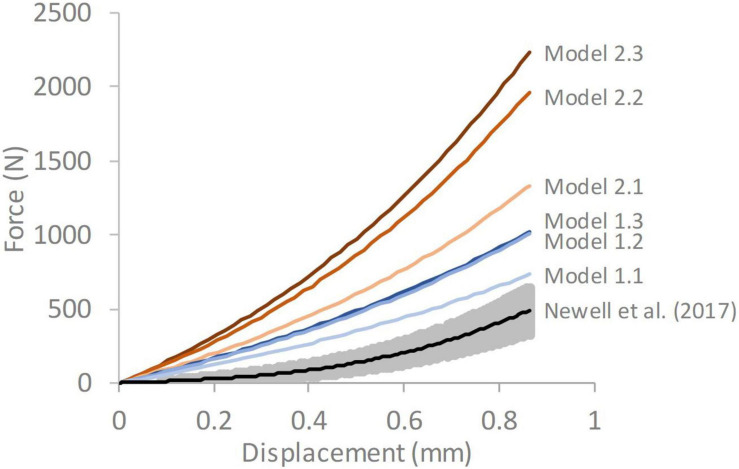
Force-displacement response of each of the six models compared against average experimental response from Newell et al. (2017) The grey shaded region on the experimental data represents ± 1 standard deviation. Model 1.1—simple endplates, linear NP-AF boundary, Model 1.2—simple endplates, curved NP-AF boundary, Model 1.3—simple endplates, segmented NP-AF boundary, Model 2.1—segmented endplates, linear NP-AF boundary, Model 2.2—segmented endplates, curved NP-AF boundary, Model 2.3—segmented endplates, segmented NP-AF boundary.

The responses obtained from all the FE models were stiffer than the average experimental response reported by Newell et al. (2017; Figure 4). A trend was seen for the stiffness to increase as the complexity of the model geometry increased. All models with segmented endplates had a greater stiffness than those with simple endplates.

**FIGURE 5:**
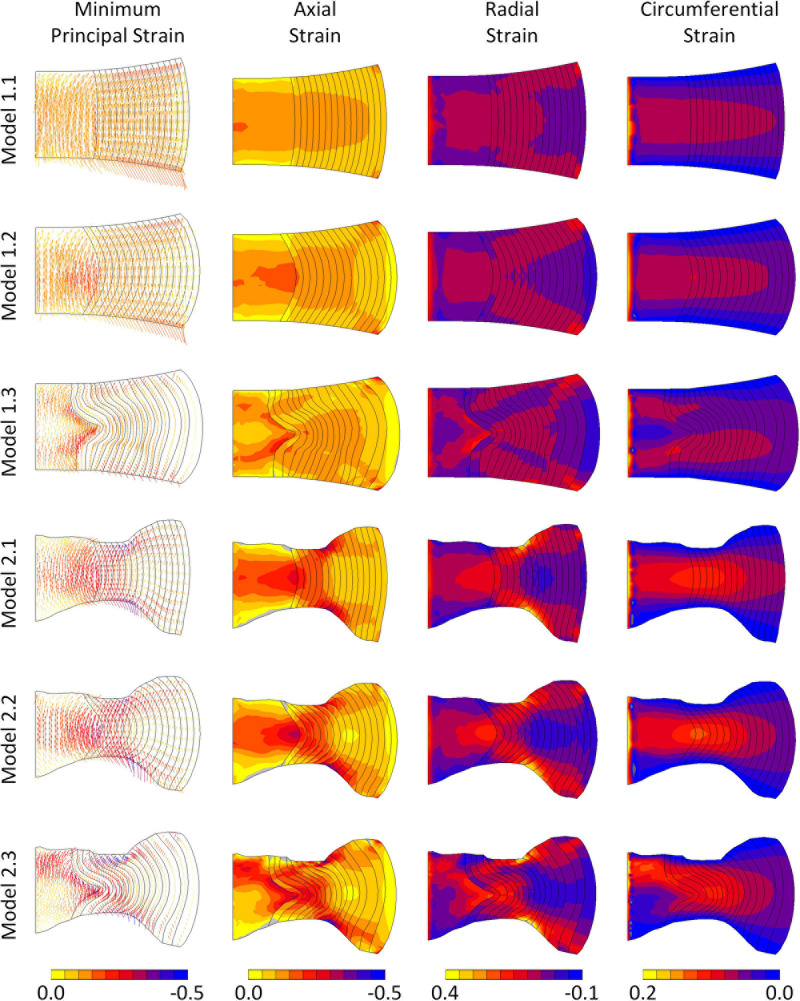
Minimum principal, axial, radial, and circumferential strain distributions in each of the six models at maximum displacement. Vectors in the left column indicate minimum principal strain directions and the colours refer to magnitudes of the minimum principal strains.

In all models the predominant direction of the minimum principal strain at maximum displacement was axial (Figure 5). Axial strains were compressive throughout the IVD in all models, and particularly high along the endplates. Peak axial strains were lowest in the models with segmented endplates, in comparison to the simple endplates (-0.22, -0.34, -0.42, -0.51, -0.64, and -1.00, for Models 1.1, 1.2, 1.3, 2.1, 2.2, and 2.3, respectively). Peak axial and radial strains were seen at locations of high endplate curvature, particularly at the mid-AF-endplate boundary of the segmented endplate models. High compressive axial strains were generally seen at the NP-AF boundary at mid-height in all models. In all models a band of high circumferential strains was seen close to the mid-height of the disc but this band veered away from mid-height in the models with segmented internal geometry (Models 1.3 and 2.3).

### Sensitivity to Altered Material Properties

**FIGURE 6:**
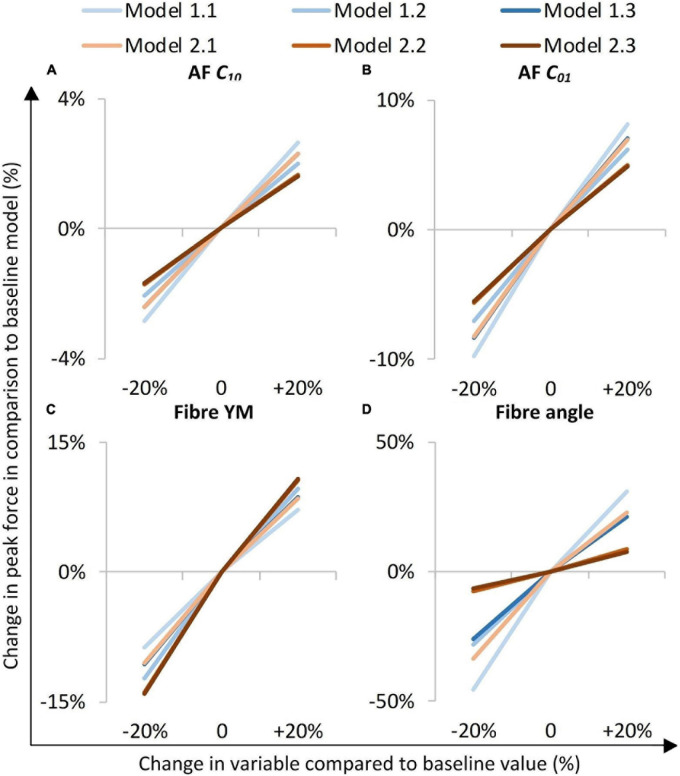
Sensitivity study overview. The y-axis represents a percentage change in peak axial force from that obtained in the initial baseline run of each of the six models when a parameter was changed by ±20%. **(A,B)** are AF ground matrix material properties, **(C,D)** are AF fibre properties.

The models were most sensitive to properties of the fibre bundles, particularly to fibre bundle angle (Figure 6D). The AF C_01_ Mooney constant had a greater effect than the AF C_10_ Mooney constant in all models (Figures 6A,B). Models with segmented endplates (2.1, 2.2, and 2.3), on average were less sensitive to changes in material properties in comparison to models with a simple endplate (Models 1.1, 1.2, and 1.3). Models 2.2 and 2.3 (segmented endplates with curved and segmented internal geometry, respectively) had similar sensitivities to all four material parameters, and both were less sensitive to the AF Mooney constants, and the fibre bundle angle, but more sensitive to the fibre bundle YM than Model 2.1 (segmented endplate, linear NP-AF boundary). Similarly, Models 1.2 and 1.3 (simple endplates with curved and segmented internal geometry, respectively) were less sensitive to the AF Mooney constants, and the fibre bundle angle, but more sensitive to the fibre bundle YM than Model 1.1 (simple endplate, linear NP-AF boundary).

### Effect of Graduated Fibre Bundle Angle

**FIGURE 7:**
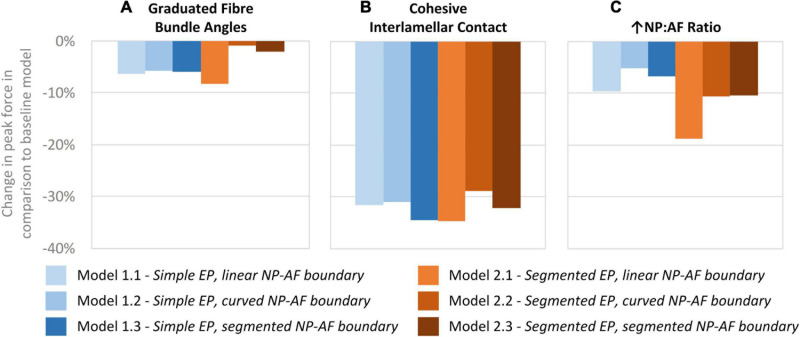
Percentage changes in peak force compared to baseline models after **(A)** graduating the fibre bundle angles, **(B)** cohesive inter-lamellar contact, and **(C)** increasing the NP:AF ratio.

A comparison of the percentage change in peak force between the baseline and varied fibre bundle angle models is shown in Figure 7A. On average, varying the fibre bundle angle resulted in a 5.0 ± 2.8% decrease in peak force in all six models in comparison to the constant fibre bundle angle models. However, Models 2.2 and 2.3 (segmented endplates with curved and segmented internal geometry, respectively) were relatively insensitive to the variation in fibre bundle angle with a percentage change of 0.9 and 2.2%, respectively, compared to 5.9–11.6% for the other models.

### Sensitivity to Modelling Inter-lamellar Behaviour With Cohesive Contact

A comparison of percentage change in peak force between the glued, and cohesive inter-lamellar contact models is shown in Figure 6B. Allowing cohesive contact caused a decrease in stiffness in comparison to the glued models for all six geometries. There were only small differences in the percentage reduction in peak force compared to the baseline models between models, with an average reduction of 32.2 ± 2.2%.

### Sensitivity to Altered NP:AF Ratio

Increasing the NP:AF ratio resulted in a reduction in peak force in all models in comparison to the baseline runs. Models 1.1 and 2.1 (simple EP, linear NP-AF boundary and segmented EP, linear NP-AF boundary) saw the greatest reduction in peak force in comparison to the curved, and segmented internal geometry models with the same endplate geometry (1.2 and 1.3, and 2.2 and 2.3, respectively). Reductions in peak force were higher in the segmented endplate models, compared to the simple endplate models (13.3 ± 4.7% vs. 7.2 ± 2.3%, respectively).

## Discussion

FE modellers are required to find a balance between geometric accuracy and keeping computational complexity low. It is therefore common to simplify model geometry to increase the likelihood of convergence. In this study ultra-high field MRI was used to obtain accurate internal geometry of an intact IVD to evaluate its effect on the response of an IVD FE model.

The six models developed in this study all had geometry based on the same MR image of a bovine IVD, however, differences were seen in terms of the location of peak strain (Figure 5), and the overall stiffness of each model (Figure 4). Maximum strains were seen at locations of high curvature in the segmented endplate models, which could not be captured in the curved endplate models. An increase in model complexity resulted in increased model stiffness, with the average peak force doubling in the segment endplate models compared to the curved endplate models (919 ± 162 N and 1,845 ± 465 N, respectively), and the average peak force being 1.44, and 1.58 times larger than the linear NP-AF boundary model (Model 1.1—simple EP, linear NP-AF boundary and Model 2.1—segmented EP, linear NP-AF boundary) in the curved (Model 1.2—simple EP, curved NP-AF boundary and segmented EP, Model 2.3—curved NP-AF boundary) and segmented (Model 1.3—simple EP, segmented NP-AF boundary and Model 2.3—segmented EP, segmented NP-AF boundary) NP-AF boundary models, respectively. The high stiffnesses in the segmented endplate models was affected by the average height being 1.22 mm shorter than the curved endplate models (7.92 vs. 9.14 mm). Even though the central, and peripheral discs heights were the same in all models the change in average height meant that the overall applied strain was greater in the segmented endplate models compared to the simple endplate models. The differences in stiffness between the segmented and simple endplate models may have been smaller had the average, rather than just central and peripheral disc heights been kept consistent between the two approaches. Additionally, a linear or curved estimation of the NP-AF boundary created idealised strain distributions (Figure 5) that neglected the effects of non-uniform lamella geometries such as variations in width and curvatures.

The force-displacement response of Model 1.1 (simple endplates, linear NP-AF boundary) was closest to the experimental data obtained by Newell et al. (2017; Figure 4). This was expected since Model 1.1’s geometry was the most similar to the models in that study in that the endplates were curved, the boundary between the NP and AF was linear, and the AF fibre YM used in this study (415 MPa) was obtained through the optimisation process described in Newell et al. (2017) that ensured a close match between experimental and numerical results. The slightly stiffer response of Model 1.1 compared the experimental data is likely due to the relatively lower NP-AF ratios, obtained from the MRIs, being used in this study (0.66:1 for Model 1.1) compared to 3.72:1 used in Newell et al. (2017). As shown in Figure 7C, increasing the NP-AF ratio decreases model stiffness, therefore had a lower NP-AF ratio been used by Newell et al. (2017), their optimised fibre stiffnesses may have been lower and therefore a closer match between experimental and numerical response may have been seen in this study.

**FIGURE 8:**
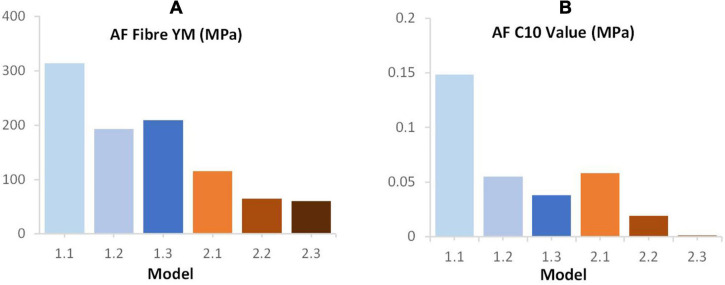
Final **(A)** AF fibre YM **(B)** C_10_ values following optimisation of these parameters in order to achieve a good fit between the computational and experimental data reported by Newell et al. (2017).

An increase in deviation from the experimental data was seen with increasing geometric complexity (Figure 4). This is likely due to all models using the same material properties, including AF fibre YM properties that were obtained in an optimisation study (Newell et al., 2017) where model geometry was most similar to Model 1.1 (simple EP, linear NP-AF boundary) in this study. Using the same optimisation algorithm described in (Newell et al., 2017) the material properties (AF fibre YM and AF C_10_) of all the models used in this study can also be optimised to obtain a close match between numerical and experimental response. This results in the optimised values shown in Figure 8 where lower AF fibre YM and AF C_10_ values were seen in the more complex models. For researchers who wish to model IVDs with accurate internal geometries it is likely that less stiff material properties are required to obtain an overall response that can be validated against experimental data. For example, the optimised AF fibre YM and AF C_10_ values for Model 2.3 (segmented endplates, segmented NP-AF boundary) were 60.63 and 0.121 MPa, respectively, which is at the lower end of the range of values that have been used in previous FE studies [44–500 MPa for AF Fibre YM and 0.0146–0.7 MPa for the AF C_10_ value (Newell et al., 2019b)].

Irrespective of geometry, the models were most sensitive to the fibre YM, and the fibre angle (on average a 10.5 ± 1.8%, and 21.6 ± 12.0% change in peak force compared to the baseline runs when adjusted by 20% of the original fibre YM and fibre angle, respectively), and relatively insensitive to the AF ground matrix properties (*C*_10_ and *C*_01_ Mooney constants—Figure 6). Although care must be taken when comparing absolute values since the sensitivity study carried out here spanned a supra-physiologic range of force these findings are similar to those of Newell et al. (2017) in terms of Fibre YM who modelled bovine discs with a curved endplate, and linear NP-AF boundary (on average a 12.9% change in peak force). Interestingly, graduating the fibre bundle angles from inner to outer AF had a relatively small effect on the overall stiffness of the model with the average peak force of all models being 5.0 ± 2.8% lower compared to baseline runs, and the peak force being just 2.2% lower in the segmented endplate, segmented NP-AF boundary model (2.3). This suggests that future studies should focus on ensuring the accuracy of the average fibre bundle angle, rather than the accuracy of fibre bundle angles in individual lamella to ensure accurate load transfer through the IVD, particularly if a segmented NP-AF boundary and segmented endplates are used. However, previous FE studies have reported sensitivity to fibre angles in terms of IVD response to torsion (Yang and O’Connell, 2017), swelling (Yang and O’Connell, 2018, 2019a), and axial stiffness (Shirazi-Adl, 1989), with the general consensus being that anatomically relevant fibre angles are important for understanding the internal stress distributions through the disc. As demonstrated in this study, and in Stadelmann et al. (2018) it is possible to use high resolution MRI to obtain these angles non-invasively. There have been two recent FE studies investigating the effect of NP:AF ratio with Yang et al. (2019) finding a positive correlation between relative NP size and IVD stiffness, and Mengoni et al. (2017) finding a negative correlation. These differences could be due to modelling bovine rather than human discs, fibres at an orientation of ± 20 degrees compared to ± 43 degrees, or modelling 20 lamella layers compared to one in the Mengoni et al. (2017) and Yang et al. (2019) studies, respectively. In this study, doubling the NP:AF ratio (equivalent to on average, increasing the NP:Disc diameter from 0.38 ± 0.01 to 0.55 ± 0.01) reduced the peak force by 10.3 ± 4.7% supporting the findings of Mengoni et al. (2017) who saw a ∼32% decrease in peak force of bovine specimens at a similar axial displacement as the maximum in this study (0.86 mm—taken from Figure 6 in Mengoni et al., 2017) when increasing the NP:Disc diameter from 0.4 to 0.6. Conversely, Yang et al. (2019) found a 23.8% increase in normalised stiffness of human IVDs under axial compression when increasing the NP:Disc diameter from 0.35 to 0.6. Depending on the method of modelling AF fibres, adjusting the NP:AF ratio can affect the total fibre volume, since IVD FE models are sensitive to fibre properties (Figure 6C) it is important to ensure that a change in total fibre volume does not affect conclusions made about altering the NP:AF ratio. In this study this was accounted for by ensuring the fibre volume was consistent before and after adjusting the NP:AF ratio.

A network of elastic fibres and collagen cross-bridges exist between lamellae in the AF which provides resistance to shearing strains (Yu et al., 2002; Pezowicz et al., 2006; Schollum et al., 2008). Most current IVD FE studies omit this inter-lamellar behaviour and either glue the boundary between lamellae (Dreischarf et al., 2014; Yang and O’Connell, 2019a, b; Yang et al., 2019), or use continuum models (Jacobs et al., 2014; Showalter et al., 2016; Newman et al., 2021). Adam et al. (2015) investigated allowing adjacent lamellae to slide freely across each other, however, experimental studies have shown that inter-lamella shearing strain is due to skewing, rather than sliding (Michalek et al., 2009; Vergari et al., 2016). A number of studies have included inter-lamellar interactions in models of several lamellar layers through the incorporation of elements or fibres in a zone between lamellar (Labus et al., 2014; Derrouiche et al., 2019; Kandil et al., 2019, 2020; Ghezelbash et al., 2021; Tamoud et al., 2021), however, the computational complexity of some of these approaches can render them impractical for modelling the whole disc (Ghezelbash et al., 2021). Mengoni et al. (2015) derived normal and tangential cohesive stiffness values that have been assigned to cohesive elements in this study to represent inter-lamellar interactions. To our knowledge this is the first time that these elements have been applied to a full IVD model and the technique proved to have potential to model the inter-lamellar behaviour more physiologically than glued contact between lamellae. Introducing this cohesive behaviour reduced the IVD stiffness (on average 32 ± 2% reduction in peak force compared to the glued baseline models – Figure 7B), which, as expected is lower than the 40% reduction shown by Adam et al. when allowing total free sliding between lamellae.

Bovine samples were modelled in this study because they are almost perfectly round (O’Connell et al., 2007; Adam et al., 2015) providing the opportunity to model axisymmetrically and thus reducing computational cost compared to full 3-D models. Beckstein et al. (2008) found the normalised stiffness of bovine IVDs to be within 12% of human IVDs suggesting similarities in mechanical properties, however, future studies to investigate how the findings in this study relate to human IVDs is required, particularly since the internal geometry of just one bovine IVD has been modelled here. Modelling one IVD was sufficient to investigate the effect of the different modelling techniques deployed in this study, however, a study on a larger population with various internal geometries, for example investigating how the internal geometry changes with degeneration and how that affects FE model behaviour would be of interest. Modelling axisymmetrically significantly reduces computational time but does not allow modes of loading other than axial compression. This meant that some geometric intricacies were simplified, and the NP was assumed to be perfectly in the centre of the IVD where in fact it was offset by approximately 1.26 mm from the centre of the disc when measured on a mid-transverse slice of the MRIs. Yang et al. (2019) found little difference in disc joint stiffness under flexion, extension, and lateral bending when changing the NP:Disc area ratio from 0.21 to 0.6, however the study did not investigate the effect of endplate and internal geometry complexity under these modes of loading.

In this study IVDs were loaded at an intermediate strain rate of 0.1/s. Previous studies have demonstrated an increase in stiffness with strain rate (Smeathers and Joanes, 1988; Yingling et al., 1997; Race et al., 2000; Kemper et al., 2007; Costi et al., 2008; Newell et al., 2017), and a recent study has demonstrated that the NP has little effect on the response of IVDs at high loading rates (Newell et al., 2019a), which differs from its function at low strain rates (Seroussi et al., 1989; Meakin and Hukins, 2000). It is therefore possible that FE models are less sensitive to geometric simplifications at higher strain rates, although further work would be required to confirm this. In this study IVDs were only subjected to pure axial compression. In order to comprehensively understand the effect of internal geometry on the outcomes of FE models, future studies should extend this analysis to investigate IVD response in combined loading and in flexion-extension, axial rotation, and lateral bending.

The complexity of the models used in this study were deliberately kept low to reduce the effects of confounding variables such as cartilage endplate properties, NP fluid phase (poroelasticity), NP swelling pressure, preload, vertebral bone properties, changes in the fibre volume fraction through the AF, and asymmetries in endplate shapes in planes other than the sagittal from which the geometry was segmented for Models 2.1—2.3 in this study. Additionally, the MRIs were obtained while the sample was well hydrated, and musculature had been removed. *In vivo*, internal geometries may change depending on posture and the time of the day and therefore the geometry used here represents the geometry at a single time point, and a single posture. Although outside the focus of this study, it is recommended that these factures, as well as those highlighted in this study are considered when developing more complex patient specific models of human discs that aim to replicate *in vivo* conditions.

## Conclusion

Geometric simplifications in FE models of IVDs create idealised load transfer that may not be physiologic. These simplifications affect model response, particularly in terms of stiffness and strain distributions, sensitivity to average fibre angles, and sensitivity to modelling inter-lamellar contact. Therefore, defining more realistic internal and external geometry of the IVD can significantly affect IVD FE model response, and it is likely that a more realistic geometry leads to a more accurate strain distribution within the IVD. It is recommended that these geometric intricacies are incorporated into IVD FE models before material properties are optimised to develop a validated model. This is particularly important if the models are being used for clinical applications such as developing repair strategies that aim to replicate the mechanical behaviour of healthy discs.

## Data Availability Statement

The original contributions presented in the study are included in the article/supplementary material, further inquiries can be directed to the corresponding author/s.

## Author Contributions

YD, UH, and NN designed the study. NB, ST, and NN designed the sequences and obtained the MRI images. YD and TR developed and ran the FE models. YD, ST, and TR analysed the data and drafted the manuscript which was edited by NN, NB, and UH. All authors approved the manuscript before submission.

## Conflict of Interest

The authors declare that the research was conducted in the absence of any commercial or financial relationships that could be construed as a potential conflict of interest. The handling editor declared a past co-authorship with one of the authors NN.
